# Bridging scales between solid mechanics and surface chemistry

**DOI:** 10.1038/s41598-022-14709-6

**Published:** 2022-06-23

**Authors:** Fabien Amiot

**Affiliations:** FEMTO-ST Institute, CNRS-UMR 6174/UBFC, 24 chemin de l’Épitaphe, 25030 Besançon, France

**Keywords:** Sensors and biosensors, Nanoparticles, Surfaces, interfaces and thin films, Mechanical engineering

## Abstract

A continuum mechanics framework is used herein to model the strains induced in a micromechanical structure by surface phenomena such as adsorption. The resulting picture significantly differs from those of a liquid under surface tension. Considering a solid isotropic elastic material, it is shown that a sphere undergoes a non uniform deformation under surface adsorption. The direction of the surface’s displacement is additionally shown to depend on both the material and the sphere’s radius. It is also shown that modeling surface effects with an elastic membrane surrounding a Cauchy elastic material, the elastic energy is usually misestimated. The reported results also reveal that the overall response of a mechanical structure to surface adsorption strongly depends, at a given scaling, of the higher-grade elastic behavior of the material.

## Introduction

It is well established that downsizing mechanical structures make their surface-over-volume ratio much larger than for usual object, so that the possibility to induce mechanical deformations using surface chemistry is significantly augmented. This scaling effect is at the heart of experimental surface science and has been initially exploited for rather fondamental surface science studies focusing on solid electrodes^1–4^. It has later been used for experimental studies of either the surface relaxation of crystals^5^ or the adsorbate-induced surface-stress^6^.

Independantly, the early 80s have seen the emergence of an increasing interest in micromechanical techniques for sensor technologies^7^, the iconic device being a cantilever structure whose deformation is measured using the optical lever technique as for atomic force microscopy (AFM)^8^. The rapid development of the latter provided a sensing platform to be tailored in order to make the cantilever bend when the targeted phenomenon occurs. These very deformable structures have thus been quickly used to renew the experimental approach of solid surfaces under various electrochemical processes^9–13^, or under simple adsorption processes^14^ so that the use of such deformable structures to reveal and measure surface stresses is widespread. The possibility to manufacture large and cheap cantilever arrays further triggered the development of micromechanical sensors^15^, so that for both fundamental and applied studies, surface stress is always analyzed or exploited through the mechanical deformation it induces on a mechanical structure. The relation between the surface chemical phenomena and the induced mechanical deformation is therefore crucial for fundamental surface science studies and for innovative applications, including those resulting from the capillary deformation of soft solids^16,17^.

One should highlight that this is a multiscale problem by nature, but that these scales are rarely addressed simultaneously. On one hand, surface phenomena are typically modeled using density function theory (DFT) simulations, which are usually limited to very few atomic layers, so that the mechanical deformation is rarely considered. Only few studies considered varying the mechanical boundary conditions, demonstrating for instance their crucial role on the energetics of adsorption^18^. On the other hand, attempts to take the mechanical structure scale into account make use of an energetic surface penalization. This surface penalization may be defined using either surface thermodynamics^19,20^ or first-principles calculations^21,22^. This implicitly assumes that the surface phenomena may be described by a membrane ascribed to deform together with the bulk material and subject to some eigenstrain. This is close in spirit to the framework developed by Gurtin and Murdoch^23,24^ to model the effect of a plastically deformed surface. Such an approach is also at the origin of the wide use of Stoney’s equation^25^ for the interpretation of the deformation of cantilever sensors : the surface and the bulk material are considered separately from the mechanical point of view, because different scales are involved : few atomic layers for surface phenomena, compared to at least hundreds of nanometers for the structure (typically for a microcantilever sensor).

Membrane-based frameworks interestingly predict a uniform and spherical strain state in nanoparticles, which is very similar to that inside a liquid drop^26,27^. This is an undeniable advantage when trying to extend to solids the well established thermodynamics of liquid surfaces. There are however now more and more evidences that the strain in nanoparticles is not uniform^28,29^, thereby suggesting that an improved mechanical description is needed in order to analyze experimental data obtained on nano-objects of various geometries^30^. Half-spaces are particular examples of structures for which mechanical deformations confined to the surface have been early evidenced^31,32^, so that an improved mechanical description should be able to describe such surface relaxation.

It has also been evidenced that the mechanical deformation significantly modifies the energetics of chemical reactions at surfaces^33–36^, which is at the heart of catalysis. This further calls for an advanced mechanical description of surfaces, which could rationalize these effects and point to the role of the material. Even though Mindlin set the basis for such an advanced description with second-strain gradient elasticity^37^, its complexity has limited its use to the investigation of size effect on the stiffness of tiny objects^38^.

## Results

**Figure 1:**
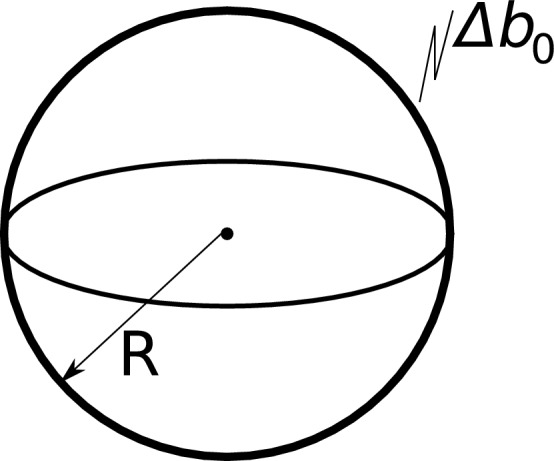
Schematic view of the addressed mechanical problem.

The first use of this continuum mechanics approach to describe the mechanical effect of surface adsorption on structures is reported herein. It is illustrated using an elastic solid sphere ${\mathscr {D}}$ made of an isotropic, centro-symmetric elastic material, whose radius is *R*. Let us denote the displacement ${\mathbf {d}}$ and assume that the material behavior is described by second-strain gradient elasticity^37^. The free energy density $\psi$ therefore reads

1 $$\begin{aligned} \psi= & {} \frac{\lambda }{2} \varepsilon _{ii}\varepsilon _{jj} + \mu \varepsilon _{ij}\varepsilon _{ij} \nonumber \\&+ a_1 \varepsilon _{ijj}\varepsilon _{ikk} + a_2 \varepsilon _{iik}\varepsilon _{kjj} + a_3 \varepsilon _{iik}\varepsilon _{jjk} + a_4 \varepsilon _{ijk}\varepsilon _{ijk} + a_5 \varepsilon _{ijk}\varepsilon _{kji} \nonumber \\&+ b_1 \varepsilon _{iijj}\varepsilon _{kkll} + b_2 \varepsilon _{ijkk}\varepsilon _{ijll} + b_3 \varepsilon _{iijk}\varepsilon _{jkll} + b_4 \varepsilon _{iijk}\varepsilon _{llkj} \nonumber \\&+ b_5 \varepsilon _{iijk}\varepsilon _{lljk} + b_6 \varepsilon _{ijkl}\varepsilon _{ijkl} + b_7 \varepsilon _{ijkl}\varepsilon _{jkli} \nonumber \\&+ c_1 \varepsilon _{ii}\varepsilon _{jjkk} + c_2 \varepsilon _{ij}\varepsilon _{ijkk} + c_3 \varepsilon _{ij}\varepsilon _{kkij} \nonumber \\&+ b_0 \varepsilon _{iijj}, \end{aligned}$$

where $\lambda$ and $\mu$ are Lamé’s coefficients, $\varepsilon _{ij}$ are the components of the classical infinitesimal strain $\varepsilon ^1$, $\varepsilon _{ijk}$ are the components of the triadic $\varepsilon ^2=\nabla \nabla {\mathbf {d}}$ (symmetric in the first two positions), and $\varepsilon _{ijkl}$ are those of $\varepsilon ^3=\nabla \nabla \nabla {\mathbf {d}}$ (symmetric in the first three positions). The higher-order elastic parameters

$$\begin{aligned} a_n, c_n, b_0\,\propto\, & {} \mu l_S^2 \\ b_{n,n>0}\,\propto\, & {} \mu l_S^4 \end{aligned}$$

make characteristic lengths $\propto l_S$ appear. These characteristic lengths (and thus the higher-grade elastic parameters) typically describe the phase’s dimensions and distribution in multi-phase materials. Besides the higher-grade quadratic terms, the presence of the linear term proportional to $\varepsilon _{iijj}$ is to be highlighted and $b_0$, which is denoted as the cohesion modulus, defines the equivalent of surface tension for solids^37^. The effect of adsorption is introduced in a strictly energetic and local way, the adsorption energy being predictable using simple approaches^39^. The displacement field in a sphere resulting from a cohesion modulus change (see Fig. 1) is computed for a large number (999) of thermodynamically admissible material parameters sets, using the closed-form solution derived in Supplementary Note A. It is found to be purely radial. The mechanical fields are driven by the length $\Lambda$ (see Supplementary Note A), which reflects the higher-grade elastic behavior of the material. For illustration purpose, it can be kept in mind that $\Lambda$ typically scales as couple of $\mathring{A}$ for crystalline materials or as few nm for amorphous materials^40,41^. The radius *R* is varied in the range $0.1 \times \Lambda<R < 100 \times \Lambda$ to reveal scaling effects, and two particular materials (denoted as ’blue’ and ’green’ material in the following), whose constitutive parameters are given in Supplementary Note B, are used to exemplify some results.

### Displacement field

**Figure 2:**
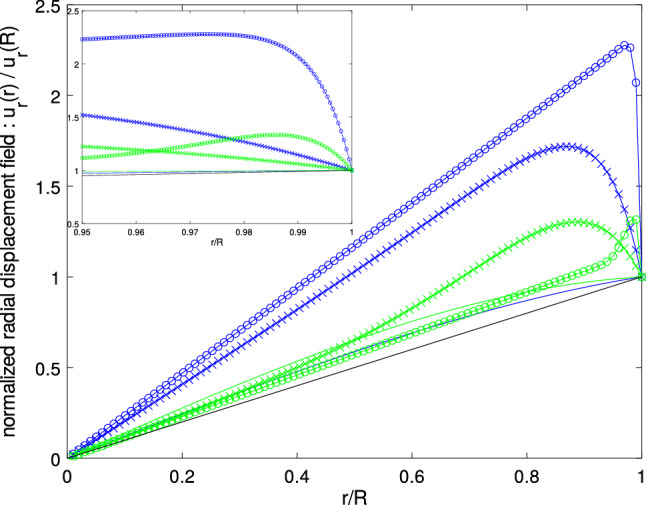
Normalized displacement field $u_r(r)/u_r(R)$ as a function of the distance from the sphere’s center *r* for the chosen materials and $R=\Lambda$ (solid line), $R=10 \times \Lambda$ (crosses), $R=100 \times \Lambda$ (circles). The normalized displacement field for a membrane-based approach is reported in black. Inset: Zoom at the sphere’s surface.

Figure 2 shows the normalized radial displacement field $u_r(r)/u_r(R)$ as a function of the current radius *r* for the materials of Supplementary Note B. $r=0$ corresponds to the sphere’s center. When the sphere radius equals the length $\Lambda$ (solid colored lines), the displacement field is almost linear with the radial position *r*, as it is anticipated using a membrane-based approach (black line, see Supplementary Note C). This strongly contrasts with the displacement fields obtained for larger values of the sphere radius, for which a strong deformation localization is observed at the surface. The radial displacement field may then be generally described as a linear function of *r*, with some possibly significant edge effect which extends over lengths scaling as the characteristic length. This further illustrates that $\Lambda$ should be seen as a cut-off length on the displacement field. This radial gradient seems consistent with some reported experimental results^28–30^. The use of the proposed framework to yield a continuous description of the displacement field at any scale is the first result of this work. It provides a mechanical description to support the interpretation of the recently reported experimental results^28–30^. The obtained expression for the displacement field simultaneously displays components at the sphere’s scale and surface components. The used framework is therefore capable to bridge the gap between the scale of surface phenomena (limited to few layers of atoms) and the structure’s scale.

### Surface displacement

**Figure 3:**
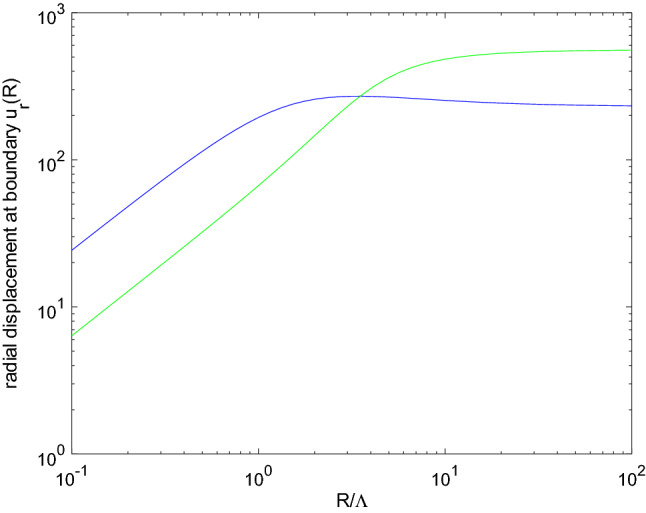
Radial displacement at the boundary $u_r(R)$ as a function of the normalized sphere’s radius $R / \Lambda$ for the blue and green materials.

The radial displacement is computed as above, and its value at the boundary $r=R$ is now considered. Figure 3 displays the radial displacement at the boundary $u_r(R)$ as a function of $R / \Lambda$ for the two considered materials. The behavior obtained with these two materials is representative of those obtained through the whole set of 999 materials. The magnitude of the radial displacement at the boundary $u_r(R)$ typically scales as *R* in the low radius regime, defined such that $R \ll \Lambda$, and this corresponds to the behavior predicted using a membrane-based approach (see Supplementary Note C). Contrarily, the large radius regime $R \gg \Lambda$ is characterized by a rather constant radial displacement at the boundary $u_r(R)$. The effect of the surface chemical change thus becomes less and less perceptible when the object’s size increases. This is absolutely consistent with a surface effect, whose impact is driven by a surface-over-volume ratio.

It is even more interesting to note that considering the same loading for all materials, and considering the large radius regime which is of more practical interest, all the materials do not undergo a deformation in the same direction. Setting $R =100 \times \Lambda$, $83\%$ of the materials are shown to expand. This means that $17\%$ contrarily shrink under the same stimulus. This exhibits the key role of the material in the mechanical response of a given structure to the same surface stimulus, far beyond its sole Cauchy stiffness. This is the second result of this work.

**Figure 4:**
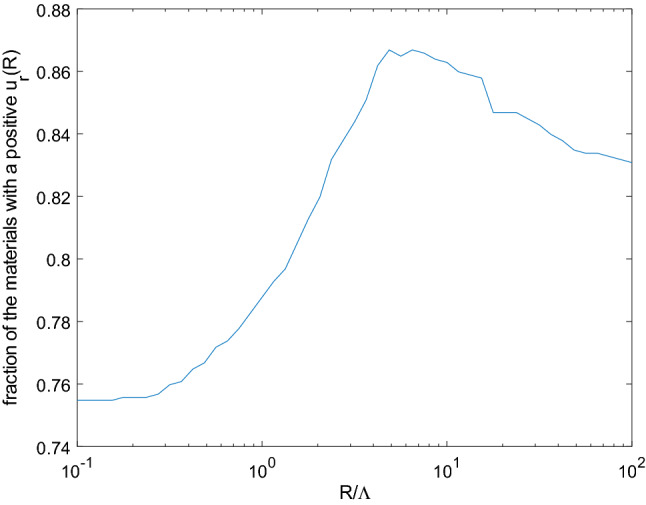
Fraction of the materials with a positive $u_r(R)$ as a function of the normalized sphere’s radius $R / \Lambda$.

Keeping the sole sign of the boundary displacement as an indicator of the response of the sphere to a surface chemical stimulus, Fig. 4 displays the fraction of the materials with a positive radial surface displacement $u_r(R)$ as a function of $R / \Lambda$. This fraction transitions from $\simeq 75 \%$ in the low radius regime ($R \ll \Lambda$) to $\simeq 83\%$ in the large radius regime in a non-monotonic way. This illustrates the role of the structure’s dimensions in its overall response. This is the third finding reported herein. This further illustrates the multiscale nature of the observed response, and thus the need for a suited mechanical modeling framework when adsorption-induced strains are under scrutiny.

## Discussion

The displacement fields obtained as closed-form solutions are further analyzed to draw practical conclusions on the modeling of surface chemo-mechanical couplings and on the involved energetics. Adsorption-induced strains are often approached by those resulting from a pre-strained membrane ascribed to deform together with the structure’s surface. The solution for the case of the sphere is recalled in Supplementary Note C. Such a description is shown to result in a radial displacement field which grows linearly with the distance to the sphere center (see Supplementary Note C.2). Comparing this result to the ones reported in Fig. 2 allows one to conclude that such membrane-based approaches yield a qualitatively correct kinematic description in the low radius regime $R \ll \Lambda$. When the sphere radius *R* increases to a range accessible to engineered structures^40–42^, this description is however found to be clearly inadequate : the multiscale nature of the fields described in Fig. 2 is lost, so that the two-regimes behavior depicted in Fig. 3 cannot be given by this more conventional approach, which yields a unique $u_r(R) \propto R$ regime.

Interestingly, the mechanical fields obtained from the membrane-based approach correspond to a uniform strain field with a purely spherical stress tensor inside the solid sphere, making it mechanically similar to a liquid drop under surface tension. This analogy has been supporting the extension of the liquid surface thermodynamics to solids^26^, but the fields reported in Fig. 2 suggest that this analogy no longer holds when the sphere radius is in the large radius regime $R \gg \Lambda$, $\Lambda$ being expected to range from $\mathring{A}$ to nm. As these results are obtained considering adsorption in a strictly energetic way, they may be extended to describe the surface relaxation of a sphere. The reported non-uniform deformation is again qualitatively consistent with experimental results focusing on a detailed picture of the atomic organization in nanoparticles^28–30^, so that the need for a continuum description of the mechanical fields such as those reported herein is highlighted.

Figures 3 and 4 evidence the role of the sphere radius on the sign and magnitude of the surface displacement. The role of the structure geometry on its overall response to a surface modification may be further illustrated by comparing the results for a very large sphere radius with those obtained in the Supplementary Note D for the surface of a half-space, extending the results initially derived in^37^. The different symmetries actually impose different strain states at the surface :

- For the half-space surface, the displacement can only be orthogonal to the surface, so that the strain is strictly uniaxial, along a direction orthogonal to the surface (see Supplementary Note D and^37^).
- Contrarily, the radial displacement in the sphere makes the strain state spherical, thus allowing for strains parallel to the surface.

This first highlights the reason why membrane-based approaches cannot describe the surface relaxation: it is impossible to find a non-vanishing solution for the half-space covered with a membrane subject to some eigenstrain, because of the incompatible displacement fields. This further highlights the limitations of membrane-based approaches in this particular case where experimental results are well established^31,32^. Second-strain gradient elasticity is contrarily already known to be able to describe such surface relaxation^37^.

The case of the plane surface furthermore cannot be seen as the sphere at the limit $R \rightarrow \infty$, and these two cases allow to assess the role of geometrical constrains. For the surface of the half-space, half of the materials in the set are found to expand, whereas the other half tends to contract towards the bulk for the same (positive) cohesion modulus change. This is in clear contrast with the case of the large sphere, for which $83\%$ of the materials are shown to expand as a response to the same surface modification, and this clearly illustrates the role of the structure geometry in its overall response to surface adsorption.

This complex interplay between the structure’s geometry and dimensions and the material’s characteristic lengths clearly hinders the use of simple relations deduced from membrane-based approaches in the interpretation of the mechanical deformations induced by adsorption phenomena. This illustrates the fact that trying to model and interpret chemo-mechanical couplings using the simple Cauchy elasticity may lead to dramatic errors or difficulties. It may also suggest a way to explain the apparently contradictory results obtained in the past with cantilever sensors^43^.

In order to quantify the transduction mechanism, the surface energy *W* (defined in Supplementary Note A.3) may be compared to the strain energy of the sphere driven by the sole Cauchy elasticity subject to the same radial displacement at the boundary $u_r(R)$. One therefore defines the indicator

$$\begin{aligned} \eta _{SSG}(R) = \frac{2 \left( 3 \lambda + 2 \mu \right) u_r^2(R) \pi R}{|W(R)|} \end{aligned}$$

as a measure of the chemo-mechanical transduction, which would be of direct interest in sensing applications. $\lambda$ and $\mu$ denote the Lamé parameters. The absolute value applied to *W* comes from the fact that *W*, as shown in Supplementary Note D, is always negative for the surface of a half-plane.

**Figure 5:**
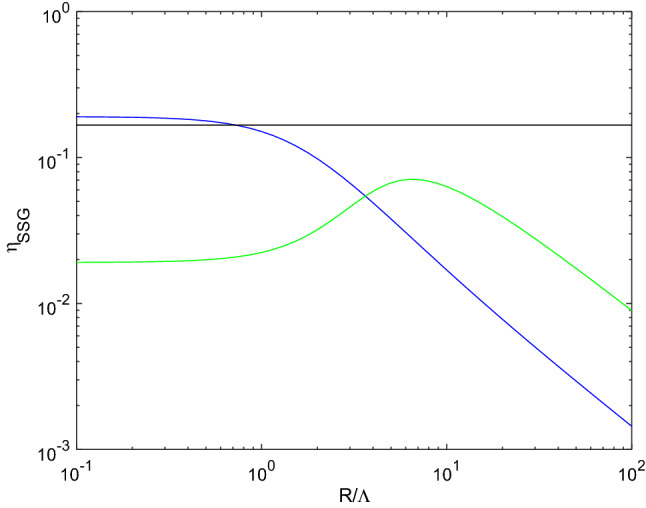
Apparent energy conversion $\eta _{SSG}(R)$ as a function of $R / \Lambda$ for the blue and green materials. Comparison with an optimal membrane-based approach (black line).

Figure 5 shows the evolution of $\eta _{SSG}$ with *R* for the materials considered in the figures above. These results are rather representative of the whole materials set. In the low radius regime $R \ll \Lambda$, $\eta _{SSG}$ tends towards a constant finite value. This is again the behavior expected from a membrane-based approach (see Supplementary Note C). Contrarily, the mechanical effect induced by the surface chemical change varies as $R^{-1}$ and vanishes when the radius turns to be much larger than $\Lambda$. This is again a clear signature of a surface effect. And again, this regime cannot be rendered by the elastic sphere surrounded by the elastic membrane, as it can be seen from the behavior of $\eta$ obtained in Supplementary Note C.2 and reported in Fig. 5: assuming a membrane-based approach, the conversion efficiency is independent on the sphere radius and cannot be larger than 1/6.

The two materials considered in Fig. 5 however display more complex and slightly different behaviors, in the sense that either $\eta _{SSG}(R)$ reaches its maximum in the low radius regime (see the blue material) or it displays a clear optimum in the intermediate regime ($R \simeq \Lambda$, see the green material). In order to quantify the capability of the material to translate a surface chemical change into a global mechanical deformation, one defines the indicator

2 $$\begin{aligned} \Psi _{SSG} = \sup _{R} \eta _{SSG}(R), \end{aligned}$$

which is found to possibly be larger than 1/6 (see the ‘blue’ material in Fig. 5). This indicator is rather specific to the considered geometrical arrangement: a sphere, whose radius change under a surface chemical change is monitored. The dependence to the sphere radius itself is however removed, so that $\Psi _{SSG}$ is only a function of the material, even though it refers to a particular geometrical arrangement.

**Figure 6:**
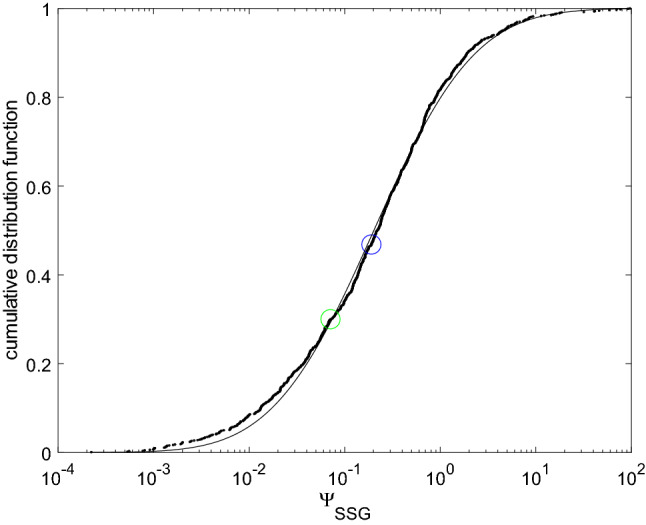
Cumulative distribution function for $\Psi _{SSG}$ and best fit using a normal distribution for $\log \left( \Psi _{SSG} \right)$ (dash-dotted line). The blue and green circles correspond to the materials chosen for illustration.

Figure 6 displays the cumulative distribution function for $\log \left( \Psi _{SSG} \right)$, computed using all the 999 materials in the dataset. The position of the ‘blue’ and ‘green’ materials is depicted using the blue and green circles, and supports the choice of these two representative materials for illustration purpose. The crucial point is the wide range a values spanned by $\Psi _{SSG}$. First, values larger than 1 are obtained, meaning that using Cauchy elasticity to analyze the deformation of the sphere can lead to dramatic errors in the estimation of the strain energy. This is the fourth reported finding.

It also turns out that $\Psi _{SSG}$ extends over more than 3 decades, thereby proving the crucial role of the material in the chemo-mechanical transduction, which therefore controls the energy conversion far beyond its Young’s modulus (which scales as unity for all the considered materials). Denoting $F^{-1}$ the inverse distribution function for $\Psi _{SSG}$, it is seen from Fig. 6 that $F^{-1}(0.9) \simeq 100 \times F^{-1}(0.1)$, so that at least 2 decades are necessary to describe $\Psi _{SSG}$. This is further confirmed by the parameters of the normal distribution fitting at best the distribution for $\log \left( \Psi _{SSG} \right)$ displayed on Fig. 6: the mean value corresponds to $\Psi _{SSG} = 0.2$, and its standard deviation corresponds to a 6.8 multiplicative factor on $\Psi _{SSG}$. The role of the higher-grade elastic parameters of the material in the chemo-mechanical coupling is therefore crucial. This is first a clear invitation, for all applications involving chemo-mechanical couplings, to carefully consider the experimental identification of the higher-grade elastic parameters. This also paves the way to the engineering of materials exhibiting optimal coupling properties, and architectured materials, which naturally display well-controlled characteristic lengths by design, are probably good candidates.

## Methods

The displacement field in a solid sphere made of an isotropic, centro-symmetric, material and subject to a cohesion modulus change has been obtained as a closed form using a Papkovich-Neuber approach in second-strain gradient elasticity. The procedure to obtain this solution is detailed in Supplementary Note A.

## Supplementary Information

Supplementary Information.

## References

[1] Bonnemay M, Bronoël G, Jonville P, Levart E (1965). Nouvelle méthode de tracé des courbes électrocapillaires sur les métaux solides. C.R. Acad. Sci. Paris.

[2] Beck TR (1969). “Electrocapillary curves” of solid metals measured by extensometer instrument. J. Phys. Chem..

[3] Fredlein RA, Damjanovic A, Bockris JOM (1971). Differential surface tension measurements at thin solid metal electrodes. Surf. Sci..

[4] Fredlein RA, Bockris JOM (1974). An electrocapillary study of the gold-perchloric acid solution interface. Surf. Sci..

[5] Martinez RE, Augustyniak WM, Golovchenko JA (1990). Direct measurement of crystal surface stress. Phys. Rev. Lett..

[6] Sander D, Ibach H (1991). Experimental determination of adsorbate-induced surface stress: Oxygen on Si(111) and Si(100). Phys. Rev. B.

[7] Seidel H, Csepregi L (1983). Three-dimensional structuring of silicon for sensor applications. Sens. Actuators.

[8] Meyer E (1992). Atomic force microscopy. Progr. Surf. Sci..

[9] Raiteri R, Butt HJ (1995). Measuring electrochemically induced surface stress with an atomic force microscope. J. Phys. Chem..

[10] Haiss W, Sass JK (1995). Adsorbate-induced surface stress at the solid electrolyte interface measured with an STM. J. Electroanal. Chem..

[11] Brunt TA, Rayment T, O’Shea SJ, Welland ME (1996). Measuring the surface stresses in an electrochemically deposited metal monolayer: Pb on Au (111). Langmuir.

[12] Ibach H, Bach CE, Giesen M, Grossman A (1997). Potential-induced stress in the solid–liquid interface: Au(111) and Au(100) in an HCl04 electrolyte. Surf. Sci..

[13] Hu K, Bard AJ (1998). In situ monitoring of kinetics of charged thiol adsorption on gold using an atomic force microscope. Langmuir.

[14] Berger R (1997). Surface stress in the self-assembly of alkanethiols on gold. Science.

[15] Lang HP (1998). A chemical sensor based on a micromechanical cantilever array for the identification of gases and vapors. Appl. Phys. A..

[16] Pericet-Cámara R, Best A, Butt H-J, Bonaccurso E (2008). Effect of capillary pressure and surface tension on the deformation of elastic surfaces by sessile liquid microdrops: An experimental investigation. Langmuir.

[17] Bico J, Reyssat E, Roman B (2018). Elastocapillarity: When surface tension deforms elastic solids. Annu. Rev. Fluid Mech..

[18] Francis MF, Curtin WA (2015). Mechanical work makes important contributions to surface chemistry at steps. Nat. Commun..

[19] Begley MR, Utz M, Komaragiri U (2005). Chemo-mechanical interactions between adsorbed molecules and thin elastic films. Jal. Mech. Phys. Solids.

[20] Amiot F (2007). A model for chemically-induced mechanical loading on MEMS. J. Mech. Mater. Struct..

[21] Dareing DW, Thundat T (2005). Simulation of adsorption-induced stress of a microcantilever sensor. J. Appl. Phys..

[22] Begley MR, Utz M (2008). Multiscale modeling of adsorbed molecules on freestanding microfabricated structures. J. Appl. Mech. Trans. ASME.

[23] Gurtin ME, Markenscoff X, Thurston RN (1976). Effect of surface stress on the natural frequency of thin crystals. Appl. Phys. Lett..

[24] Gurtin ME, Murdoch AI (1978). Surface stress in solids. Int. J. Solids Struct..

[25] Stoney G (1909). The tension of metallic films deposited by electrolysis. Proc. R. Soc. Lond. Ser. A.

[26] Vermaak JS, Mays CW, Kuhlmann-Wilsdorf D (1968). On surface stress and surface tension: I. Theoretical considerations. Surf. Sci..

[27] Mays CW, Vermaak JS, Kuhlmann-Wilsdorf D (1968). On surface stress and surface tension. II. Determination of the surface stress of gold. Surf. Sci..

[28] Marks LD (1985). Inhomogeneous strains in small particles. Surf. Sci..

[29] Marks LD (1994). Experimental studies of small particle structures. Rep. Prog. Phys..

[30] Krayzman V (2020). Local structural distorsions and failure of the surface-stress “Core-Shell” model in Brookite titania nanorods. Chem. Mater..

[31] Germer LH, MacRae AU (1962). Surface reconstruction caused by adsorption. PNAS.

[32] Germer LH, MacRae AU (1962). Adsorption of hydrogen on (110) Nickel surface. J. Chem. Phys..

[33] Gsell M, Jakob P, Menzel D (1998). Effect of substrate strain on adsorption. Science.

[34] Kibler LA, El-Aziz AM, Hoyer R, Kolb DM (2005). Tuning reaction rates by lateral strain in a palladium monolayer. Angew. Chem. Int. Ed..

[35] Weissmüller J, Viswanath RN, Kibler LA, Kolb DM (2011). Impact of surface mechanics on the reactivity of electrodes. Phys. Chem. Chem. Phys..

[36] Weissmüller J (2019). Adsorption-strain coupling at solid electrodes. Curr Opin Chem. Eng..

[37] Mindlin RD (1965). Second-gradient theory of strain and surface tension in linear elasticity. Int. J. Solids Struct..

[38] Cordero NM, Forest S, Busso EP (2016). Second-strain gradient elasticity of nano-objects. J. Mech. Phys. Solids.

[39] Gao W (2020). Determining the adsorption energies of small molecules with the intrinsic properties of adsorbates and substrates. Nat Commun..

[40] Maranganti R, Sharma P (2007). Length scales at which classical elasticity breaks down for various materials. Phys. Rev. Lett..

[41] Maranganti R, Sharma P (2007). A novel atomistic approach to determine strain-gradient elasticity constants: Tabulation and comparison for various metals, semiconductors, silica, polymers and the (ir) relevance for nanotechnologies. J. Mech. Phys. Solids.

[42] Jakata K, Every AG (2008). Determination of the dispersive elastic constants of the cubic crystals Ge, Si. GaAs and InSb. Phys. Rev. B.

[43] Boisen A, Dohn S, Keller SS, Schmid S, Tenje M (2011). Cantilever-like micromechanical sensors. Rep. Progr. Phys..

