# Supplementary material for: Bridging scales between solid mechanics and surface chemistry
Source: Sci Rep. 2022 Jun 23;12:10665. doi: 10.1038/s41598-022-14709-6 (PMC9226078; doi:10.1038/s41598-022-14709-6)
Supplement: Supplementary file 1 — Supplementary Information. [file 41598_2022_14709_MOESM1_ESM.pdf]

Bridging scales between solid mechanics and surface chemistry : Supplementary Notes

F. Amiot

FEMTO-ST Institute, CNRS-UMR 6174 / UBFC

24 chemin de l'Épitaphe, 25030 Besançon, France

fabien.amiot@femto-st.fr

June 17, 2022

A Solution for a sphere

One considers a sphere $\mathcal{D}$ made of an isotropic, centro-symmetric elastic material, whose radius is R. Let us denote the displacement $\mathbf{d}$ and assume that the material behavior is described by second-strain gradient elasticity [1]. The free energy density ψ therefore reads

$$\begin{aligned}
\psi &= \frac{\lambda}{2}\epsilon_{ii}\epsilon_{jj} + \mu\epsilon_{ij}\epsilon_{ij} \\
&+a_1\epsilon_{ijj}\epsilon_{ikk} + a_2\epsilon_{iik}\epsilon_{kjj} + a_3\epsilon_{iik}\epsilon_{jjk} + a_4\epsilon_{ijk}\epsilon_{ijk} + a_5\epsilon_{ijk}\epsilon_{kji} \\
&+b_1\epsilon_{iijj}\epsilon_{kkll} + b_2\epsilon_{ijkk}\epsilon_{ijll} + b_3\epsilon_{iijk}\epsilon_{jkll} + b_4\epsilon_{iijk}\epsilon_{llkj} \\
&+b_5\epsilon_{iijk}\epsilon_{lljk} + b_6\epsilon_{ijkl}\epsilon_{ijkl} + b_7\epsilon_{ijkl}\epsilon_{jkli} \\
&+c_1\epsilon_{ii}\epsilon_{jjkk} + c_2\epsilon_{ij}\epsilon_{ijkk} + c_3\epsilon_{ij}\epsilon_{kkij} \\
&+b_0\epsilon_{iijj} \qquad (1)
\end{aligned}$$

where λ and μ are Lamé's coefficients, ϵ_{ij} are the components of the classical infinitesimal strain ϵ^1, ϵ_{ijk} are the components of the triadic $\epsilon^2 = \nabla\nabla\mathbf{d}$ (symmetric in the first two positions), and ϵ_{ijkl} are those of $\epsilon^3 = \nabla\nabla\nabla\mathbf{d}$ (symmetric in the first three positions). The higher-order elastic parameters

$$\begin{aligned}
a_n, c_n, b_0 &\propto \mu l_S^2 \\
b_{n,n>0} &\propto \mu l_S^4
\end{aligned}$$

make characteristic lengths $\propto l_S$ appear. These characteristic lengths (and thus the higher-grade elastic parameters) typically describe the phase's dimensions and distribution in multi-phase materials. Besides the higher-grade

quadratic terms, the presence of the linear term proportional to ϵ_{iijj} is to be highlighted and b_0, which is denoted as the cohesion modulus, defines the equivalent of surface tension for solids [1]. The purpose of this Section is to derive the closed-form expression of the displacement field when the surface chemistry is modified, that is when the cohesion modulus b_0 is subject to a change Δb_0.

A.1 Governing equations

Any solution $\mathbf{u}$ of the displacement-equation of equilibrium can be expressed as [1]

$$\mathbf{u} = \mathbf{B} - (l_{21}^2 + l_{22}^2 - l_{21}^2 l_{22}^2 \nabla^2) \nabla\nabla \cdot \mathbf{B} - \frac{1}{2} \nabla \left(D_{11}^2 D_{12}^2 - k^{-1} \right) \left(\mathbf{r} \cdot D_{21}^2 D_{22}^2 \mathbf{B} + B_0 \right) \tag{2}$$

where $\mathbf{r}$ is the position vector. $\mathbf{B}$ and B_0 vector and scalar potentials to be determined. Assuming there is no body force,

$$k = \frac{\lambda + 2\mu}{\mu} \tag{3}$$

$$D_{11}^2 D_{12}^2 \nabla^2 B_0 = 0 \tag{4}$$

$$D_{21}^2 D_{22}^2 \nabla^2 \mathbf{B} = \mathbf{0} \tag{5}$$

For the sake of brevity, the operators

$$D_{ij}^2 = 1 - l_{ij}^2 \nabla^2 \tag{6}$$

have been introduced, with the lengths l_{ij} satisfying

$$\begin{aligned}
2(\lambda+2\mu)l_{1j}^2 &= \bar{a}-2\bar{c}\pm\sqrt{(\bar{a}-2\bar{c})^2-4\bar{b}(\lambda+2\mu)}\,;\; j=1,2 && (7)\\
2\mu l_{2j}^2 &= \bar{a'}-c_3\pm\sqrt{(\bar{a'}-c_3)^2-4\bar{b'}\mu} && (8)\\
\bar{a} &= 2(a_1+a_2+a_3+a_4+a_5) && (9)\\
\bar{b} &= 2(b_1+b_2+b_3+b_4+b_5+b_6+b_7) && (10)\\
\bar{c} &= c_1+c_2+c_3 && (11)\\
\bar{a'} &= 2(a_3+a_4) && (12)\\
\bar{b'} &= 2(b_5+b_6) && (13)
\end{aligned}$$

One thus has to find the scalar field B_0 and the vector field $\mathbf{B}$ satisfying Eqs (4) and (5) together with the boundary conditions. Because of the symmetry of the problem, one makes use of a spherical coordinates system $(\mathbf{e_r}, \mathbf{e}_\theta, \mathbf{e}_\phi)$ and these fields depend only on $r = |\mathbf{r}|$. As the operators commute in (4), B_0 reads

$$B_0 = B_0(r) = B_0^0(r) + B_0^1(r) + B_0^2(r) \tag{14}$$

with

$$\begin{aligned}
\nabla^2 B_0^0 &= 0 && (15)\\
(1-l_{11}^2\nabla^2)B_0^1 &= 0 && (16)\\
(1-l_{12}^2\nabla^2)B_0^2 &= 0 && (17)
\end{aligned}$$

The solution is straightforward :

$$
\begin{aligned}
B_0^0 &= -\frac{A_{00}}{r} + C_{00} && (18)\\
B_0^1 &= A_{01}\frac{e^{-\frac{r}{l_{11}}}}{r} + B_{01} l_{11}\frac{e^{\frac{r}{l_{11}}}}{r} && (19)\\
B_0^2 &= A_{02}\frac{e^{-\frac{r}{l_{12}}}}{r} + B_{02} l_{12}\frac{e^{\frac{r}{l_{12}}}}{r} && (20)
\end{aligned}
$$

where A_{00}, C_{00}, A_{01}, B_{01}, A_{02} and B_{02} are to be set from the boundary conditions. Similarly, $\mathbf{B}$ reads

$$\mathbf{B} = \mathbf{B^0} + \mathbf{B^1} + \mathbf{B^2} \tag{21}$$

with

$$
\begin{aligned}
\nabla^2 \mathbf{B^0} &= 0 && (22)\\
(1 - l_{21}^2 \nabla^2)\mathbf{B^1} &= 0 && (23)\\
(1 - l_{22}^2 \nabla^2)\mathbf{B^2} &= 0 && (24)
\end{aligned}
$$

$\mathbf{B^0}$ is thus found to be purely radial :

$$\mathbf{B^0} = \left(\frac{A_{r0}}{r^2} + B_{r0} r\right)\mathbf{e_r} \tag{25}$$

and similarly,

$$
\begin{aligned}
\mathbf{B^1} &= \left(A_{r1} i\sqrt{\frac{2}{\pi}}\left(-\frac{l_{21}^2}{r^2}\sinh\left(\frac{r}{l_{21}}\right)+\frac{l_{21}}{r}\cosh\left(\frac{r}{l_{21}}\right)\right)\right. \\
&\left.+B_{r1}\sqrt{\frac{2}{\pi}}\left(\frac{l_{21}^2}{r^2}\cosh\left(\frac{r}{l_{21}}\right)-\frac{l_{21}}{r}\sinh\left(\frac{r}{l_{21}}\right)\right)\right)\mathbf{e_r} \qquad (26)\\
\mathbf{B^2} &= \left(A_{r2} i\sqrt{\frac{2}{\pi}}\left(-\frac{l_{22}^2}{r^2}\sinh\left(\frac{r}{l_{22}}\right)+\frac{l_{22}}{r}\cosh\left(\frac{r}{l_{22}}\right)\right)\right. \\
&\left.+B_{r2}\sqrt{\frac{2}{\pi}}\left(\frac{l_{22}^2}{r^2}\cosh\left(\frac{r}{l_{22}}\right)-\frac{l_{22}}{r}\sinh\left(\frac{r}{l_{22}}\right)\right)\right)\mathbf{e_r} \qquad (27)
\end{aligned}
$$

A_{r1}, B_{r1}, A_{r2} and B_{r2} are again constants to be set from the boundary conditions. The resulting displacement field is then expressed as a function of the constants introduced above using Eq. (2).

The displacement field is found to possibly have a component along $\mathbf{e}_\phi$ which depends only on A_{r1}, B_{r1}, A_{r2} and B_{r2}. For the sake of symmetry, one therefore imposes in the following

$$A_{r1} = B_{r1} = A_{r2} = B_{r2} = 0 \qquad (28)$$

which makes the displacement purely radial, with

$$
\begin{aligned}
2ku_r r^2 &= -A_{01}\frac{r+l_{11}}{l_{11}}e^{-\frac{r}{l_{11}}} - A_{02}\frac{r+l_{12}}{l_{12}}e^{-\frac{r}{l_{12}}} \\
&+B_{01}(r-l_{11})e^{\frac{r}{l_{11}}} + B_{02}(r-l_{12})e^{\frac{r}{l_{12}}} \\
&+(1-k)A_{00} - A_{r0}(1-3k) + 2B_{r0}r^3 \qquad (29)
\end{aligned}
$$

As a consequence, the solution is driven by l_{11} and l_{12}, so that results are displayed as a function of Λ

$$\Lambda = \frac{l_{11} + l_{12}}{2} \tag{30}$$

As seen from Eq. (6), the lengths l_{ij} and thus Λ should be seen as cut-off lengths on the displacement fields. Imposing that $\mathbf{B}$ and B_0 remain bounded inside the sphere yields

$$A_{r0} = 0 \tag{31}$$

$$A_{00} = A_{01} + B_{01}l_{11} + A_{02} + B_{02}l_{12} \tag{32}$$

A Taylor expansion of the radial component u_r of the displacement field at $r = 0$ reads

$$\begin{aligned} u_r \underset{r=0}{\sim} & \frac{-1}{2r^2}\left(l_{12}B_{02} + l_{11}B_{01} + A_{02} + A_{01}\right) \\ & + \frac{k^{-1}}{4l_{11}^2 l_{12}^2}\left(l_{12}^2\left(A_{01} + l_{11}B_{01}\right) + l_{11}^2\left(A_{02} + l_{12}B_{02}\right)\right) + \cdots \end{aligned} \tag{33}$$

One therefore simultaneously imposes

$$A_{01} + l_{11}B_{01} = 0 \tag{34}$$

$$A_{02} + l_{12}B_{02} = 0 \tag{35}$$

so that the displacement at the center of the sphere is zero and the radial displacement field depends only on the 3 unknowns, B_{01}, B_{02} and B_{r0}. The

radial displacement reads :

$$
\begin{aligned}
u_r &= B_{r0}k^{-1}r \\
&+k^{-1}\frac{B_{01}e^{\frac{r}{l_{11}}}}{2r^2}\left((r+l_{11})\,e^{-2\frac{r}{l_{11}}}+r-l_{11}\right) \\
&+k^{-1}\frac{B_{02}e^{\frac{r}{l_{12}}}}{2r^2}\left((r+l_{12})\,e^{-2\frac{r}{l_{12}}}+r-l_{12}\right)
\end{aligned} \tag{36}
$$

and these 3 unknowns are to be set from the boundary conditions. In case the lengths l_{11} and l_{12} are complex, $l_{11} = \tilde{l_{12}}$ (where $\tilde{}$ denotes the complex conjugate) so the constants A_{01}, A_{02}, B_{01} and B_{02} are to be sought as complex numbers satisfying

$$A_{02} = \tilde{A_{01}} \tag{37}$$

$$B_{02} = \tilde{B_{01}} \tag{38}$$

to ensure a real valued displacement. Setting

$$A_{01} = a_r + ia_i \tag{39}$$

$$B_{01} = b_r + ib_i \tag{40}$$

$$l_{11} = s + il \tag{41}$$

the displacement field is conveniently recast as

$$
\begin{aligned}
2ku_r r^2 &= 2\left(\sin\left(\frac{lr}{s^2+l^2}\right)\left(-e^{\frac{rs}{s^2+l^2}}\left(b_i(s-r)+b_r l\right)+e^{\frac{-rs}{s^2+l^2}}\frac{a_i(s^2+rs+l^2)-a_r lr}{s^2+l^2}\right)\right. \\
&\left.+\cos\left(\frac{lr}{s^2+l^2}\right)\left(-e^{\frac{rs}{s^2+l^2}}\left(b_r(s-r)-b_i l\right)+e^{\frac{-rs}{s^2+l^2}}\frac{-a_r(s^2+rs+l^2)-a_i lr}{s^2+l^2}\right)\right) \\
&+(1-k)A_{00}-A_{r0}(1-3k)+2B_{r0}r^3
\end{aligned} \tag{42}
$$

and a similar development follows.

A.2 Boundary conditions

Defining the associated generalized stresses as

$$\begin{aligned} \tau^1 &= \frac{\partial\psi}{\partial\epsilon^1} \\ \tau^2 &= \frac{\partial\psi}{\partial\epsilon^2} \\ \tau^3 &= \frac{\partial\psi}{\partial\epsilon^3} \end{aligned}$$

and denoting $\mathbf{n}$ the unit outward normal at the surface, the boundary conditions read

$$\mathbf{t^3} = \mathbf{0} \ \text{ for } r = R \tag{43}$$

$$\mathbf{t^2} = \mathbf{0} \ \text{ for } r = R \tag{44}$$

$$\mathbf{t^1} = \mathbf{0} \ \text{ for } r = R \tag{45}$$

where $\mathbf{t^3}$, $\mathbf{t^2}$ and $\mathbf{t^1}$ are the generalized tractions [1]:

$$\mathbf{t^3} = \mathbf{nnn} \,\vdots\, \tau^3 \tag{46}$$

$$\mathbf{t^2} = \mathbf{nn} : (\tau^2 - \nabla\cdot\tau^3) + \mathbf{n}\cdot\big(\mathbf{L}\cdot(\mathbf{n}\cdot\tau^3)\big) + \mathbf{L}\cdot(\mathbf{nn}:\tau^3) \tag{47}$$

$$\begin{aligned} \mathbf{t^1} = {} & \mathbf{n}\cdot\big(\tau^1 - \nabla\cdot\tau^2 + \nabla\nabla : \tau^3\big) \\ & + \mathbf{L}\cdot\left(\mathbf{n}\cdot(\tau^2 - \nabla\cdot\tau^3) + \mathbf{L}\cdot(\mathbf{n}\cdot\tau^3) - (\overset{s}{\nabla}\mathbf{n})\cdot(\mathbf{nn}:\tau^3)\right) \end{aligned} \tag{48}$$

and, for the sphere,

$$\overset{s}{\nabla} = (\mathcal{I} - \mathbf{e_r} \otimes \mathbf{e_r}) \cdot \nabla \tag{49}$$

$$\mathbf{L} = \frac{2}{r}\mathbf{e_r} - \overset{s}{\nabla} \tag{50}$$

Eqs. (43), (44) and (45) are found to yield an equation only along $\mathbf{e_r}$, so that a total of 3 linear equations is available to set the 3 unknowns. This linear system reads

$$\begin{bmatrix} s_{11} & s_{12} & k^{-1}(3c_1 + c_2 + c_3) \\ s_{21} & s_{22} & \frac{11k^{-1}}{3R}(3c_1 + c_2 + c_3) \\ s_{31} & s_{32} & \frac{k^{-1}}{3R^2}(3R^2(3\lambda + 2\mu) + 33c_1 + 11c_2 + 11c_3) \end{bmatrix} \mathbf{U} = \begin{bmatrix} -1 \\ -\frac{11}{3R} \\ -\frac{11}{3R^2} \end{bmatrix} \Delta b_0 \tag{51}$$

where

$$\mathbf{U}^t = [B_{01}, B_{02}, B_{r0}]^t \tag{52}$$

and the detailed expressions of the s_{ij} are given in A.4. The solution for a unit Δb_0 then reads

$$B_{01} = -\frac{k^{-1}(3Rs_{22} - 11s_{12})(3\lambda + 2\mu)}{3DR} \tag{53}$$

$$B_{02} = \frac{k^{-1}(3Rs_{21} - 11s_{11})(3\lambda + 2\mu)}{3DR} \tag{54}$$

$$B_{r0} = -\frac{3R^2\left(s_{21}s_{32} - s_{22}s_{31}\right) - 11Rs_{11}s_{32} + 11Rs_{12}s_{31} + 11s_{11}s_{22} - 11s_{12}s_{21}}{3DR^2} \tag{55}$$

$$\begin{aligned} D = {} & \frac{k^{-1}}{3R^2}\Big(s_{11}\left(s_{22}\left(3R^2\left(3\lambda + 2\mu\right) + 11\left(c_3 + c_2 + 3c_1\right)\right) - 11R\left(c_3 + c_2 + 3c_1\right)s_{32}\right) \\ & -s_{12}\left(s_{21}\left(3R^2\left(3\lambda + 2\mu\right) + 11\left(c_3 + c_2 + 3c_1\right)\right) - 11R\left(c_3 + c_2 + 3c_1\right)s_{31}\right)\Big) \\ & +k^{-1}\left(c_3 + c_2 + 3c_1\right)\left(s_{21}s_{32} - s_{22}s_{31}\right) \end{aligned} \tag{56}$$

and the displacement field is uniquely determined, provided that the system's determinant D does not vanish.

A.3 Surface energy

Let us denote W the integral of the strain energy ψ (defined by Eq.(1)) over the sphere $\mathcal{D}$:

$$W = \int_{\mathcal{D}} \psi(r) dV \tag{57}$$

As already shown in [1], in the absence of mechanical loading, W reduces to

$$W = \frac{b_0}{2} \int_{\mathcal{D}} \nabla^2 \nabla \cdot \mathbf{u} dV = \frac{b_0}{2} \int_{\sigma} \mathbf{n} \cdot \nabla \nabla \cdot \mathbf{u} dS \tag{58}$$

where σ denotes the surface of the sphere $\mathcal{D}$. For the considered loading, the surface (input) energy may thus be expressed as a linear combination of the unknowns :

$$\begin{aligned} \frac{W k l_{11}^2 l_{12}^2}{\pi \Delta b_0} &= -B_{02} l_{11}^2 e^{-\frac{R}{l_{12}}} \left(l_{12} e^{\frac{2R}{l_{12}}} - \left(R e^{\frac{2R}{l_{12}}} + l_{12} + R \right) \right) \\ &\quad - B_{01} l_{12}^2 e^{-\frac{R}{l_{11}}} \left(l_{11} e^{\frac{2R}{l_{11}}} - \left(R e^{\frac{2R}{l_{11}}} + l_{11} + R \right) \right) \end{aligned} \tag{59}$$

for the real case. It may be seen that it does not depend on B_{r0}, which is expected since the Cauchy deformation mode (proportional to B_{r0}) satisfies

$\mathbf{n} \cdot \nabla\nabla \cdot \mathbf{u} = 0$ on σ. For the complex case, one obtains

$$
\begin{aligned}
\frac{Wk\left(s^2+l^2\right)^2}{2\pi\Delta b_0} \quad = &\\
& b_r e^{-\frac{Rs}{s^2+l^2}} \left(l \left(\left(s\left(s-2R\right)+l^2\right) e^{\frac{2Rs}{s^2+l^2}} + s\left(s+2R\right)+l^2 \right) \sin\left(\frac{Rl}{s^2+l^2}\right) \right. \\
& \qquad \left. + \left(R\left(s^2-l^2\right)\left(1+e^{\frac{2Rs}{s^2+l^2}}\right) + s\left(s^2+l^2\right)\left(1-e^{\frac{2Rs}{s^2+l^2}}\right)\right)\cos\left(\frac{Rl}{s^2+l^2}\right)\right) \\
& -b_i e^{-\frac{Rs}{s^2+l^2}} \left(\left(R\left(s^2-l^2\right)\left(1-e^{\frac{2Rs}{s^2+l^2}}\right) + s\left(s^2+l^2\right)\left(1+e^{\frac{2Rs}{s^2+l^2}}\right)\right)\sin\left(\frac{Rl}{s^2+l^2}\right) \right. \\
& \qquad \left. + l\left(\left(s\left(s-2R\right)+l^2\right)e^{\frac{2Rs}{s^2+l^2}} - \left(s\left(s+2R\right)+l^2\right)\right)\cos\left(\frac{Rl}{s^2+l^2}\right)\right)
\end{aligned} \tag{60}
$$

A.4 Coefficients

$$
\begin{aligned}
s_{1i}kR^5e^{-\frac{R}{l_{1i}}} &= -R\left(1+e^{-\frac{2R}{l_{1i}}}\right)\times \\
&\quad \left(R^2\left(\frac{2\left(2\left(b_7+b_6\right)+b_5+b_4+b_3+b_2\right)}{l_{1i}^2}+c_3+c_2\right)+24\left(b_7+b_6\right)\right) \\
&\quad +\frac{l_{1i}}{2}\left(1-e^{-\frac{2R}{l_{1i}}}\right)\times \\
&\quad \left(\frac{R^4\bar{c}}{l_{1i}^2}+2\left(R^2\left(\frac{4b_2l_{1i}^2+R^2\bar{b}}{2l_{1i}^4}+c_3+c_2\right)\right.\right. \\
&\quad \left.\left.+2\left(\frac{R^2\left(6\left(b_7+b_6\right)+b_5+b_4+b_3\right)}{l_{1i}^2}+12\left(b_7+b_6\right)\right)\right)\right) \qquad (61)
\end{aligned}
$$

$$
\begin{aligned}
s_{2i}kR^6e^{-\frac{R}{l_{1i}}} &= \frac{-R}{2}\left(1+e^{-\frac{2R}{l_{1i}}}\right)\times \\
&\quad \left(R^2\left(\frac{R^2\left(-2a_1l_{1i}^2+\bar{b}\right)}{l_{1i}^4}+\frac{R^2\bar{c}+46\left(b_7+b_6\right)}{l_{1i}^2}\right.\right. \\
&\quad \left.-\frac{-74\left(b_5+b_4\right)-65b_3-56b_2+3R^2\left(\bar{a}-2a_1\right)}{3l_{1i}^2}+\frac{37c_3+28c_2}{3}\right) \\
&\quad \left.+12\left(18\left(b_7+b_6\right)-R^2\left(a_5+a_4\right)\right)\right) \\
&\quad +\frac{l_{1i}}{2}\left(1-e^{-\frac{2R}{l_{1i}}}\right)\times \\
&\quad \left(R^2\left(\frac{-6R^2\left(3\left(a_5+a_4\right)+a_3+a_2+a_1\right)}{3l_{1i}^2}\right.\right. \\
&\quad +\frac{R^2\left(42\left(b_7+b_6\right)+38\left(b_5+b_4\right)+35b_3+32b_2+28b_1\right)}{3l_{1i}^4} \\
&\quad +\frac{R^2\left(16c_2+14c_1\right)+354\left(b_7+b_6\right)+74\left(b_5+b_4\right)+65b_3+56b_2}{3l_{1i}^2} \\
&\quad \left.+c_3\left(\frac{19R^2}{3l_{1i}^2}+\frac{37}{3}\right)+\frac{28c_2}{3}\right) \\
&\quad \left.+12\left(18\left(b_7+b_6\right)-R^2\left(a_5+a_4\right)\right)\right) \qquad (62)
\end{aligned}
$$

$$
\begin{aligned}
s_{3i} k R^7 e^{-\frac{R}{l_{1i}}} \quad = \quad & \frac{-R}{2}\left(1+e^{-\frac{2R}{l_{1i}}}\right)\times \\
& \left(R^2\left(R^2\left(4\mu+\frac{8\left(-a_1 l_{1i}^2+b_7+b_6+b_5+b_4\right)+7b_3+6\left(b_2+b_1\right)}{l_{1i}^4}\right)\right.\right. \\
& -\frac{-104\left(b_5+b_4\right)-80b_3-56b_2+6R^2\left(5\left(a_5+a_4\right)+4\left(a_3+a_2\right)\right)}{3l_{1i}^2} \\
& \left.+\frac{52c_3}{3}+\frac{28c_2}{3}+\frac{R^2\left(6c_3+5c_2\right)+3\left(R^2c_1+24\left(b_7+b_6\right)\right)}{l_{1i}^2}\right) \\
& \left.+42\left(8\left(b_7+b_6\right)-R^2\left(a_5+a_4\right)\right)\right) \\
& +l_{1i}\left(1-e^{-\frac{2R}{l_{1i}}}\right)\times \\
& \left(\frac{R^4}{l_{1i}^4}\left(2\mu l_{1i}^4+\left(\frac{13c_3}{2}+\frac{14c_2}{3}+\frac{10c_1}{3}-4\left(3\left(a_5+a_4\right)+a_3+a_2+a_1\right)\right)l_{1i}^2\right.\right. \\
& \left.+13\left(b_7+b_6\right)+11\left(b_5+b_4\right)+\frac{55b_3}{6}+\frac{22b_2}{3}+\frac{20b_1}{3}\right) \\
& +R^2\left(\frac{276\left(b_7+b_6\right)+52\left(b_5+b_4\right)+40b_3+28b_2}{3l_{1i}^2}+\frac{26c_3}{3}+\frac{14c_2}{3}\right) \\
& \left.+21\left(8\left(b_7+b_6\right)-R^2\left(a_5+a_4\right)\right)\right) \qquad (63)
\end{aligned}
$$

B Material parameters for the reported results

999 material parameters sets have been obtained by randomly generating parameters sets with $l_S = 1 \times 10^{-3}$ and keeping those yielding a positive definite stiffness tensor, under the constrains $\lambda > 0$ and $\mu > 0$ [2]. These parameters sets can be scaled to physical ones by setting physical l_S values [3, 4, 5]. It should first be outlined that the vast majority ($\sim 92\%$) of the parameters sets yield complex-valued l_{ij} lengths. This is known to correspond to interaction stiffnesses quickly decreasing with the distance between interacting particles in lattice materials [1]. Two specific materials, which yield real-valued (blue) and complex-valued (green) characteristic lengths l_{11} and l_{12} and whose parameters are displayed in the Table below, have been chosen among these to illustrate the results.

All the material parameters sets are freely available at [6].

Parameter	'blue' material	'green' material
λ (a.u.)	0.23450	0.89866
μ (a.u.)	0.32954	0.57411
$a_1 \times l_S^{-2}$	0.405576	0.422018
$a_2 \times l_S^{-2}$	0.455259	0.406719
$a_3 \times l_S^{-2}$	0.054128	0.346389
$a_4 \times l_S^{-2}$	0.360096	0.128259
$a_5 \times l_S^{-2}$	0.197339	0.084851
$b_1 \times l_S^{-4}$	0.381855	-0.072306
$b_2 \times l_S^{-4}$	0.312357	0.075082
$b_3 \times l_S^{-4}$	-0.147836	-0.185854
$b_4 \times l_S^{-4}$	0.083769	0.354568
$b_5 \times l_S^{-4}$	0.362985	0.296555
$b_6 \times l_S^{-4}$	0.439393	0.239330
$b_7 \times l_S^{-4}$	0.489509	0.385830
$c_1 \times l_S^{-2}$	-0.084471	0.118333
$c_2 \times l_S^{-2}$	-0.297773	0.448119
$c_3 \times l_S^{-2}$	-0.469331	-0.034367
$l_{11} \times l_S^{-1}$	1.01	1.08 - 0.61 i
$l_{12} \times l_S^{-1}$	2.04	1.08 + 0.61 i

Table 1: Materials parameters for the 'blue' and 'green' materials used to illustrate the results.

C Solution for Cauchy elasticity and a shell

C.1 General solution

As a reference solution, we recall herein the solution for the isotropic elastic sphere surrounded by an elastic shell subject to eigenstrain (see Fig. 1). Because of the symmetry of the problem, the solution is sought as a radial displacement field $\mathbf{u} = u_r(r)\mathbf{e_r}$ depending only on the distance from the sphere center r.

In the general case the medium is subjected to an isotropic uniform eigen-

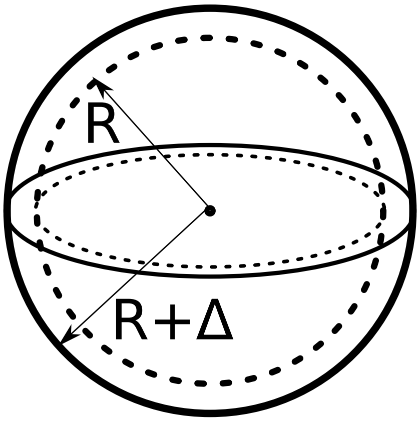

Figure 1: The isotropic elastic sphere surrounded by an elastic shell subjected to eigenstrain.

strain $\epsilon_0\mathcal{I}$, the stress components read

$$\sigma_{rr} = \lambda\left(\frac{\mathrm{d}u_r}{\mathrm{d}r} + 2\frac{u_r}{r} - \epsilon_0\right) + 2\mu\left(\frac{\mathrm{d}u_r}{\mathrm{d}r} - \frac{\epsilon_0}{3}\right) \tag{64}$$

$$\sigma_{\theta\theta} = \lambda\left(\frac{\mathrm{d}u_r}{\mathrm{d}r} + 2\frac{u_r}{r} - \epsilon_0\right) + 2\mu\left(\frac{u_r}{r} - \frac{\epsilon_0}{3}\right) = \sigma_{\phi\phi} \tag{65}$$

where (λ, μ) are the Lamé coefficients of the material, so that the equilibrium condition

$$\frac{\mathrm{d}\sigma_{rr}}{\mathrm{d}r} + \frac{2}{r}\left(\sigma_{rr} - \sigma_{\theta\theta}\right) = 0 \tag{66}$$

yields

$$\frac{\mathrm{d}^2u_r}{\mathrm{d}r^2} + \frac{2}{r}\frac{\mathrm{d}u_r}{\mathrm{d}r} - \frac{2u_r}{r^2} = 0 \tag{67}$$

As a consequence, u_r reads

$$u_r = C_1 r + \frac{C_2}{r^2} \tag{68}$$

where C_1 and C_2 are constants to be set from the boundary conditions. This general form is now particularized considering the bulk is free of any eigen-strain, and that the surrounding shell is subjected to an initial eigenstrain $\epsilon_0 \mathcal{I}$. Keeping the displacement bounded makes the displacement field sought as

$$u_r = C_{1B} r \quad \text{in the bulk} \tag{69}$$

$$u_r = C_{1S} r + \frac{C_{2S}}{r^2} \quad \text{in the shell} \tag{70}$$

and C_{1B}, C_{1S} and C_{2S} are to be set from the boundary conditions. It should be outlined that the solution (69) yields a purely spherical stress state in the bulk. The interface between the bulk and the shell being located at $r = R$, the displacement continuity reads

$$C_{1B} R = C_{1S} R + \frac{C_{2S}}{R^2} \tag{71}$$

The continuity of the traction at the interface solely imposes the continuity of σ_{rr}

$$C_{1B}\left(3\lambda + 2\mu\right) = \left(3\lambda_S + 2\mu_S\right)\left(C_{1S} - \frac{\epsilon_0}{3}\right) - 4\mu_S \frac{C_{2S}}{R^3} \tag{72}$$

This radial stress also has to vanish at the outer boundary of the shell, whose thickness is Δ

$$\left(3\lambda_S + 2\mu_S\right)\left(C_{1S} - \frac{\epsilon_0}{3}\right) - 4\mu_S \frac{C_{2S}}{(R+\Delta)^3} = 0 \tag{73}$$

The conditions (71), (72) and (73) thus define a linear system which is solved to set C_{1B}, C_{1S} and C_{2S}. The energy transfer from the shell to the bulk is assessed by comparing the strain energy in the bulk $E_{\mathcal{B}}$

$$\begin{aligned} E_{\mathcal{B}} &= \frac{1}{2}\int_{\mathcal{B}} \sigma_{rr}\epsilon_{rr} + 2\sigma_{\theta\theta}\epsilon_{\theta\theta} dV \\ &= 2\left(3\lambda + 2\mu\right) C_{1B}^2 \pi R^3 \end{aligned} \tag{74}$$

to the strain energy $E_{\mathcal{S}}$ introduced in the shell through the eigenstrain

$$\begin{aligned} E_{\mathcal{S}} &= \frac{1}{2}\int_{\mathcal{S}} \sigma_{rr}\epsilon_{rr} + 2\sigma_{\theta\theta}\epsilon_{\theta\theta} dV \\ &= \frac{2\epsilon_0^2}{9}\left(3\lambda_S + 2\mu_S\right)\pi\left((R+\Delta)^3 - R^3\right) \end{aligned} \tag{75}$$

It is thus possible to define the conversion efficiency η such that

$$\eta = \frac{E_{\mathcal{B}}}{E_{\mathcal{S}}} = \frac{9\left(3\lambda + 2\mu\right) C_{1B}^2}{\epsilon_0^2\left(3\lambda_S + 2\mu_S\right)} \frac{R^3}{(R+\Delta)^3 - R^3} \tag{76}$$

C.2 Asymptotic behavior

In order to provide a description for surface effects, one considers herein the $\frac{\Delta}{R} \to 0$ limit. Comparing Eqs (72) and (73), it is clear that the necessary condition for strain energy to be transferred to the bulk is that the product $\mu_S \Delta R^{-1}$ does not vanish. It is thus assumed hereafter that

$$\mu_S \frac{\Delta}{R} = M \tag{77}$$

and this scaling behavior is considered to compute the limit of the above-defined quantities. Considering that λ_S does not vary with $\frac{\Delta}{R}$ yields

$$C_{1B} \underset{\Delta/R=0}{\rightarrow} \frac{4M\epsilon_0}{12M + 3\left(3\lambda + 2\mu\right)} \tag{78}$$

$$\eta \underset{\Delta/R=0}{\rightarrow} \frac{8\left(3\lambda + 2\mu\right)M}{3\left(\left(3\lambda + 2\mu\right) + 4M\right)^2} \tag{79}$$

The radial displacement at the boundary $u_r(R) = C_{1B}R$ is then found to scale as R and the conversion efficiency is found to be independent on the sphere radius. η displays a maximum for

$$M_{opt} = \frac{3\lambda + 2\mu}{4} \tag{80}$$

which corresponds to $\eta = \frac{1}{6}$, whatever the considered material.

D Second-strain gradient elasticity at the plane surface of a solid

The calculation provided by Mindlin [1] is extended herein to the case of complex-valued lengths l_{ij} and consequences of the positive definite character of the stiffness operator are given. Considering the half-plane $\mathcal{H} : x \geq 0$, the symmetry imposes that the displacement field reads

$$\mathbf{u} = u_x(x)\mathbf{e_x} \tag{81}$$

so that the displacement has to satisfy

$$\left(1 - l_{11}^2 \frac{\mathrm{d}^2}{\mathrm{d}x^2}\right)\left(1 - l_{12}^2 \frac{\mathrm{d}^2}{\mathrm{d}x^2}\right)\frac{\mathrm{d}^2 u_x}{\mathrm{d}x^2} = 0 \tag{82}$$

with the constitutive equations

$$\tau_{xx} = (\lambda + 2\mu)\frac{\mathrm{d}u_x}{\mathrm{d}x} + \bar{c}\frac{\mathrm{d}^3 u_x}{\mathrm{d}x^3} \tag{83}$$

$$\tau_{xxx} = \bar{a}\frac{\mathrm{d}^2 u_x}{\mathrm{d}x^2} \tag{84}$$

$$\tau_{xxxx} = b_0 + \bar{c}\frac{\mathrm{d}u_x}{\mathrm{d}x} + \bar{b}\frac{\mathrm{d}^3 u_x}{\mathrm{d}x^3} \tag{85}$$

and the boundary conditions

$$\tau_{xxxx} = 0 \text{ at } x = 0 \tag{86}$$

$$\tau_{xxx} - \frac{\mathrm{d}\tau_{xxxx}}{\mathrm{d}x} = 0 \text{ at } x = 0 \tag{87}$$

$$\tau_{xx} - \frac{\mathrm{d}\tau_{xxx}}{\mathrm{d}x} + \frac{\mathrm{d}^2\tau_{xxxx}}{\mathrm{d}x^2} = 0 \text{ at } x = 0 \tag{88}$$

The general solution of Eq. (82) vanishing when x is very large reads

$$u_x = A_1 e^{-\frac{x}{l_{11}}} + A_2 e^{-\frac{x}{l_{12}}} \tag{89}$$

where the constants A_1 and A_2 are to be set from the boundary conditions. If the lengths l_{11} and l_{12} are complex valued, then again setting

$$l_{11} = s + il \tag{90}$$

makes the solution sought as

$$u_x = e^{-\frac{sx}{s^2+l^2}} \left(A_c \cos\left(\frac{lx}{s^2+l^2}\right) + A_s \sin\left(\frac{lx}{s^2+l^2}\right)\right) \tag{91}$$

where the constants A_c and A_s are to be set from the boundary conditions. For both the real and complex cases, the boundary conditions set yields only two equations. For the real case :

$$\begin{bmatrix} l_{12}^2\left(l_{11}^2 + \frac{\bar{c}}{\lambda+2\mu}\right) & l_{11}^2\left(l_{12}^2 + \frac{\bar{c}}{\lambda+2\mu}\right) \\ l_{12}\left(l_{12}^2 + \frac{\bar{c}}{\lambda+2\mu}\right) & l_{11}\left(l_{11}^2 + \frac{\bar{c}}{\lambda+2\mu}\right) \end{bmatrix} \begin{bmatrix} A_1 \\ A_2 \end{bmatrix} = \begin{bmatrix} 0 \\ \frac{b_0 l_{11} l_{12}}{\lambda+2\mu} \end{bmatrix} \tag{92}$$

and for the complex case :

$$\begin{bmatrix} s\left(\frac{\bar{b}(3l^2-s^2)}{(s^2+l^2)^3} + \frac{\bar{c}}{s^2+l^2}\right) & l\left(\frac{\bar{b}(3s^2-l^2)}{(s^2+l^2)^3} + \frac{\bar{c}}{s^2+l^2}\right) \\ -\frac{\bar{b}(s^2-2ls-l^2)(s^2+2ls-l^2)}{(s^2+l^2)^4} - \frac{(\bar{c}-\bar{a})(s^2-l^2)}{(s^2+l^2)^2} & \frac{\bar{b}4ls(s^2-l^2)}{(s^2+l^2)^4} + \frac{2ls(\bar{c}-\bar{a})}{(s^2+l^2)^2} \end{bmatrix} \begin{bmatrix} A_c \\ A_s \end{bmatrix} = \begin{bmatrix} -b_0 \\ 0 \end{bmatrix} \tag{93}$$

, from which the solutions for the displacement field follow. Substituting this

solution into the definition of the surface energy (58) yields

$$\frac{W}{S} = \frac{b_0^2\left(l_{12}^2 - l_{11}^2\right)}{2\left(\left(l_{11}^2 + \frac{\bar{c}}{\lambda+2\mu}\right)^2 l_{12} - l_{11}\left(l_{12}^2 + \frac{\bar{c}}{\lambda+2\mu}\right)^2\right)(\lambda + 2\mu)} \tag{94}$$

for the real case and

$$\frac{W}{S} = -\frac{b_0^2\bar{b}s}{\bar{c}(\bar{c}-\bar{a})\left(s^2+l^2\right)^2 + 2\bar{b}\bar{c}(s^2-l^2) + \bar{a}\bar{b}(s^2+l^2) + \bar{b}^2} \tag{95}$$

for the complex case. These expressions are rewritten as

$$\frac{W}{S} = -\frac{b_0^2\left(l_{12} + l_{11}\right)}{2\left(l_{11}l_{12}\left(l_{11}^2 + l_{12}^2 + \frac{2\bar{c}}{\lambda+2\mu}\right) + \left(l_{11}^2 l_{12}^2 - \left(\frac{\bar{c}}{\lambda+2\mu}\right)^2\right)\right)(\lambda + 2\mu)} \tag{96}$$

for the real case and

$$\frac{W}{S} = -\frac{b_0^2 s}{\bar{b} - \frac{\bar{c}^2}{\lambda+2\mu} + \bar{a}\sqrt{\frac{\bar{b}}{\lambda+2\mu}}} \tag{97}$$

for the complex case. It is however easily seen from the positive definiteness of the quadratic part of ψ (see Eq.(1)) that

$$\lambda + 2\mu > 0 \tag{98}$$

$$\bar{a} > 0 \tag{99}$$

$$\bar{b} > 0 \tag{100}$$

$$\bar{b} - \frac{\bar{c}^2}{\lambda + 2\mu} > 0 \tag{101}$$

As a consequence, all the terms appearing in Eq. (96) and Eq. (97) are

positive, thereby proving that $\frac{W}{S}$ is always negative for a plane surface.

References

[1] Mindlin, R.D. Second-gradient theory of strain and surface tension in linear elasticity. *Int. J. Solids Structures* **1,** 417-438 (1965).

[2] Amiot, F. Constitutively optimal governing equations for higher-order elastic beams. *Eur. J. Mech. -A/Solids* **86,** 104195 (2021).

[3] Maranganti, R., Sharma, P. Length scales at which classical elasticity breaks down for various materials. *Phys. Rev. Lett.* **98,** 195504 (2007).

[4] Maranganti, R., Sharma, P. A novel atomistic approach to determine strain-gradient elasticity constants: tabulation and comparison for various metals, semiconductors, silica, polymers and the (ir) relevance for nanotechnologies. *J. Mech. Phys. Solids* **55,** 1823-1852 (2007).

[5] Jakata, K., Every, A.G. Determination of the dispersive elastic constants of the cubic crystals Ge, Si, GaAs and InSb. *Phys. Rev. B* **77,** 174301 (2008).

[6] Amiot, F. Second-strain gradient elasticity parameters for centrosymmetric materials. *Zenodo* `https://zenodo.org/record/6283226` (2022).
